# Scalable colored Janus fabric scheme for dynamic thermal management

**DOI:** 10.1016/j.isci.2024.110948

**Published:** 2024-09-13

**Authors:** Sijie Pian, Zhuning Wang, Chengtao Lu, Peixuan Wu, Qikai Chen, Xu Liu, Yaoguang Ma

**Affiliations:** 1 State Key Laboratory for Extreme Photonics and Instrumentation, College of Optical Science and Engineering, Intelligent Optics and Photonics Research Center, Jiaxing Research Institute, International Research Center for Advanced Photonics, ZJU–Hangzhou Global Scientific and Technological Innovation Center, Zhejiang University, Hangzhou 310027, China

**Keywords:** Applied sciences, Biomaterials

## Abstract

The art of passive thermal management lies in effectively mitigating heat stress by manipulating the optical spectra of target objects. However, a significant obstacle remains in finding a structure that can seamlessly adapt to diverse thermal environments. In response to this challenge, we posit that Janus fabrics have unique advantages for multi-scene applications when carefully engineered. A Janus fabric with an upper side exhibiting a 92% solar reflectivity and a 94% emissivity, along with a lower side possessing an infrared emissivity below 30% could enable energy savings at a large scale. It outperforms commercial products in terms of energy-saving efficiency under different climate conditions. Furthermore, the scalable manufacturing compatibility and outstanding performance make the Janus structure a promising avenue for diverse passive thermal management scenarios.

## Highlights

• Dual-mode radiative thermal management fabric based on fiber and woven structure
• Simultaneous coloring of cooling/thermal insulation sides based on multilayer design
• Easily compatible with scalable manufacturing processes
• Positive thermal management and energy saving effect in different climate conditions

## Graphical abstract

**Figure:**
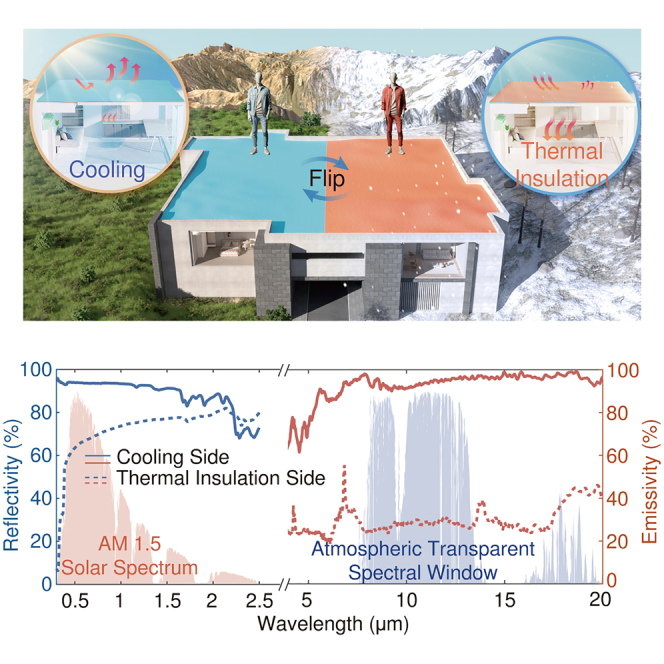

## Introduction

The severity of extreme weather conditions is having an increasingly detrimental effect on thermal management, thereby endangering human beings, and amplifying the load and energy usage of Heating, Ventilation, and Air Conditioning (HVAC) systems. This global issue is further exacerbated by the presence of two billion air conditioning units and 40% of households’ demand for heating, resulting in the extensive consumption of electricity, natural gas, coal, and other energy sources.^1^ Consequently, the annual release of CO_2_ emissions amounts to billions of tons. Efforts are being made worldwide to foster the development of renewable and low-carbon energy systems in order to realize the goal of achieving Net Zero Emissions (NZE). In this context, harnessing passive technologies to achieve cost-effective energy reduction becomes crucial for sustainable development. Recently, the engineering of surface radiative properties has emerged as a promising approach for personal thermal management (PTM) and energy savings in domains such as buildings and transportation. For instance, by rejecting most solar radiation while maximizing heat radiation within the atmospheric transparent spectral window (ATSW, 8–13 μm), positive cooling power can be achieved at ambient temperatures without any additional energy consumption.^2^^,^^3^^,^^4^^,^^5^^,^^6^ This concept has led to successful implementations of advanced low-cost radiative cooling designs, including coatings,^7^^,^^8^^,^^9^^,^^10^ films,^11^^,^^12^^,^^13^ plates,^14^^,^^15^ textiles,^16^^,^^17^^,^^18^^,^^19^ and among others which have demonstrated significant cooling effects with added protection effects. However, the monotonous optical properties of such demonstrations limited their effectiveness either to hot or cold environments, resulting in heating or cooling penalties in opposite climates.

**Figure 1:**
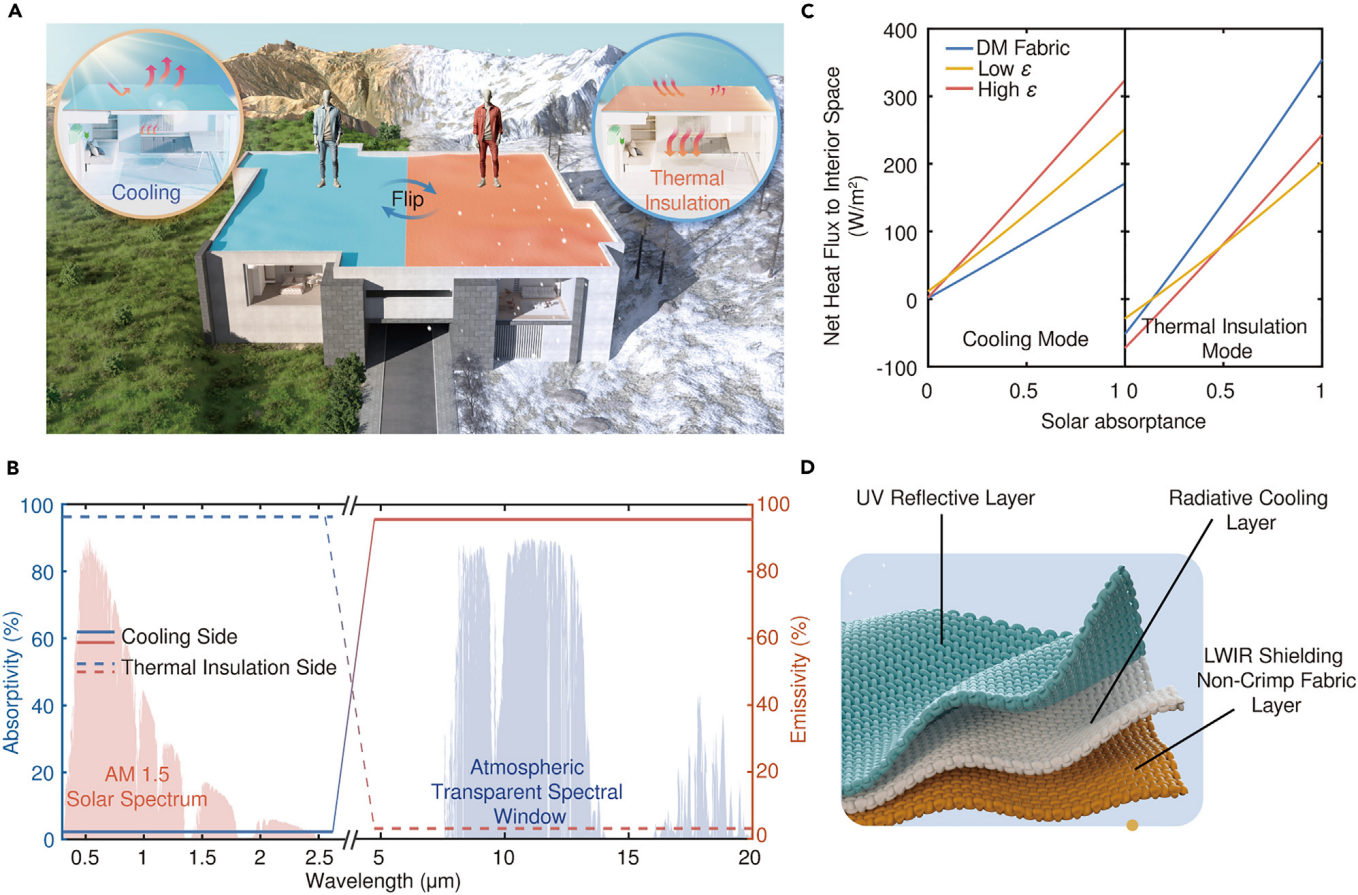
Principle, structure, and manufacturing process of the DM fabric (A) Schematic of the DM fabric for dual-mode thermal management. (B) Optical performance for ideal Janus structures. (C) Net heat flux to thermally managed objects versus solar absorptance for different types of fabrics. In most cases, the DM fabric maintains the lowest/highest internal heat load in the cooling/thermal insulation mode. Low *ε*/high *ε*, fabric with low/high LWIR emissivity on both sides. (D) Structure and principles of the DM fabric.

To optimize thermal management effects across various climate zones and seasons, innovative strategies such as thermochromic materials and Janus structures have been proposed.^20^^,^^21^^,^^22^^,^^23^^,^^24^^,^^25^^,^^26^^,^^27^ These approaches aim to control the longwave infrared (LWIR) thermal radiation within the ATSW band, effectively shutting it down (even utilizing solar radiation for heating purposes) when cooling is unnecessary, exhibiting remarkable spectral control capabilities and adaptability to different environmental conditions. Among them, the fabrication of thermochromic materials at a large scale is hindered by the high cost and requirement for cleanroom-level facilities, which affects their uniformity and overall quality. The lack of material consistency limits their effectiveness in practical applications due to inadequate tunability. The use of thermochromic microcapsules to achieve temperature adaptive regulation of solar radiation absorption has been proposed recently, which significantly reduces the difficulty of fabrication and alleviates the coloration problem. These results have achieved extraordinary dynamic spectral control capabilities and excellent thermal management effects.^28^ Due to material properties, the spectral control range is often limited to one of the solar radiation bands and the LWIR bands, so there is still room for research in exploring the simultaneous regulation of two bands. Furthermore, once the components of such materials are determined, their transition temperatures will be fixed. This makes it difficult to flexibly respond to different climates and the customized needs of different consumers. In addition, the porous film-based moisture transfer technique can effectively realize broadband dynamic regulation of solar radiation and LWIR, and exhibits astonishing dual-mode thermal management performance, providing a feasible solution for energy-saving temperature regulation.^29^ On the other hand, Janus structures are closest to reality due to their simplicity for real-life applications, ease of preparation, and user-friendly operation. This kind of design offers switchable dual-mode thermal management, making them highly desirable for applications in both indoor thermal management (ITM)^30^^,^^31^^,^^32^^,^^33^ and PTM^24^^,^^34^^,^^35^^,^^36^ as shown in Figure 1A. Utilizing some tiny mechanical energy, this type of structure can quickly switch thermal management modes according to the actual needs of the users. It does not require the introduction of additional media (such as liquid) during mode switching and is more portable and adaptable in applications such as personal thermal management and high-frequency climate change environments with large temperature differences between day and night. However, most existing Janus structures proposed are non-woven structures such as films and plates, limiting their versatility. In contrast, fiber-based fabric structures hold significant advantages in terms of breathability, comfort, and flexibility, enabling broader applicability across multiple scenarios.^18^^,^^19^^,^^20^^,^^37^^,^^38^^,^^39^^,^^40^ By finding materials and micro-nano structures that can fulfill the requirements of broadband, asymmetric spectral regulation while maintaining wearability, inherent surface unevenness of fabrics at the optical scale can be overcome to realize efficient spectral regulation. Furthermore, additional design and manufacturing techniques could enable the harmonious integration of fabric coloring while minimizing the potential effects of dyes on spectral performance.^41^^,^^42^^,^^43^

Here, we demonstrate the effectiveness of a dual-mode Janus fabric (DM fabric) with distinct thermal management properties switchable with mechanical operations. Optical and thermal characterization has demonstrated its efficacy in achieving superior radiative cooling capabilities as well as providing outstanding thermal insulation properties, thereby positioning it as a promising solution for both PTM and ITM applications covering most scenarios encountered in daily thermal management. Macroscopically, the DM fabric takes the form of a three-dimensional woven multilayer structure, graded spatially to accommodate structures in various scales, incorporating nanostructures, fiber structures, and fabric spatial configuration. The pivotal role played by nanostructures enables functions such as exceptional reflectivity and captivating fabric coloring while regulating spectral performance within the LWIR band. As a result, it achieves diverse LWIR emissivity on its front and back sides leading to extraordinary thermal regulation effects such as radiative cooling or thermal insulation.

## Results

### Optical properties required for efficient climate accommodations and aesthetic requirements

An ideal Janus structure should have two typical optical properties as shown in Figure 1B. This unique feature enables achieving unparalleled heat load reduction or insulation capabilities for various cooling or thermal insulation applications. When the temperature of the fabric rises due to solar absorption, the low emissivity of the Janus structure’s inner surface in cooling mode can suppress the thermal radiation to the indoor space. On the other hand, the high emissivity of the Janus structure’s inner surface in thermal insulation mode can effectively enhance the thermal radiation to the indoor space, thereby improving the utilization efficiency of absorbed solar energy. Serving as the interface between thermal management objects and the external environment, the asymmetric spectrum design of the DM fabric offers a distinct advantage over competing products that possess identical optical properties on both sides (Figure 1C and optical characterization in method details). A Janus fabric can be designed with a multilayered configuration consisting of an ultraviolet (UV) reflective layer, a radiative cooling layer, and an LWIR shielding non-crimp fabric^44^ layer as shown in Figure 1D. On opposite sides of this fabric, the LWIR emissivity is maximized and minimized respectively.

**Figure 2:**
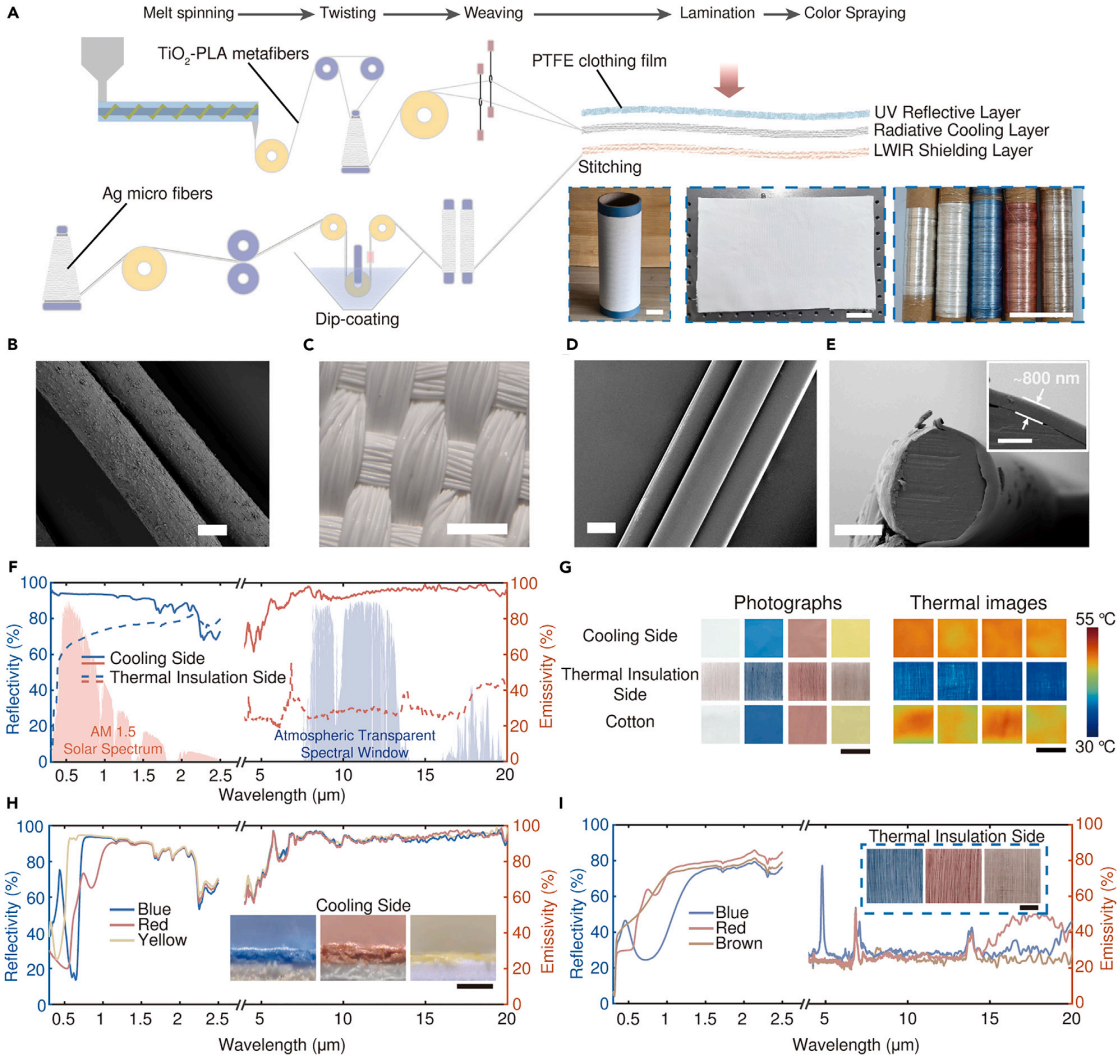
Optical properties of the DM fabric (A) Fabrication process of the DM fabric. The insets show the TiO_2_-PLA metafibers, the woven radiative cooling layer, and the fabricated LWIR shielding fibers (blank, white, blue, red, and brown) with lengths of hundreds of meters in sequence from left to right. Scale bars, 40 mm. (B) SEM image of the prepared TiO_2_-PLA metafibers. Scale bar, 20 μm. (C) Micrograph of the prepared radiative cooling fabric layer. The fabric is opaque white, indicating its strong scattering of light. Scale bar, 500 μm. (D) SEM image of the surfaces of the Ag-PE core-shell fibers. Scale bar, 20 μm. (E) SEM image of the cross section of the Ag-PE core-shell fiber. Scale bar, 10 μm. Inset shows an enlarged view of the fiber cross section. Scale bar, 2 μm. PE shell with a thickness of ∼800 nm has been successfully coated on the surface of the Ag filament. (F) Measured reflectivity and emissivity spectra of the white DM fabric. AM, air mass. (G) Photographs and thermal images of the DM fabrics and cotton fabrics with different colors. Scale bars, 25 mm. (H) Measured reflectivity and emissivity of the colored DM fabrics’ cooling sides in the solar radiation band. The insets show the optical micrographs of colored layers. Scale bar, 100 μm. (I) Measured reflectivity and emissivity of the colored DM fabrics’ thermal insulation sides in the solar radiation band. The insets show the optical micrographs of corresponding samples. Scale bar, 5 mm.

By employing industrial manufacturing techniques (Figure 2A), we have scaled up the production of the Janus fabric. The UV reflective layer consists of a non-woven clothing film structure of polytetrafluoroethylene (PTFE), containing numerous cross-linked nanometer-level fibers and particles dispersed within (Figure S1). As shown in Figures 2B and 2C, the radiative cooling layer is woven from polylactic acid (PLA) fibers doped with titanium oxide (TiO_2_) nanoparticles that strongly scatter sunlight (Figure S2). PTFE exhibits very low UV absorption due to its simple structure (only C-C and C-F bonds). The woven textile beneath provides broadband reflection for solar radiation (0.4–2.5 μm) (Figures S3 and S4). The emissivity could also be further enhanced due to increased optical path length caused by absorption and scattering of micron-scale hybrid fibers^45^ as well as gradient refractive index anti-reflection effect caused by undulating fabric surface,^7^^,^^46^ resulting in elevated broadband LWIR emissivity compared to pure PLA fibers alone as shown in Figure S5.

Instead of using reflective coatings or metal foils,^24^^,^^34^ the outward-facing non-crimp fabric layer is composed of metal-core/polyethylene (PE) -shell fibers showing efficient LWIR shielding. Here, as a means of demonstration, Ag micron filaments were chosen as the cores. By utilizing angle-weighted scattering efficiency (Q_aw_ = scattering efficiency × (1 - *g*), where *g* is the asymmetric parameter),^47^ we quantitatively analyzed backward scattering efficiencies of fibers made from different materials (Figure S6). The core diameter was selected at 25 μm to ensure flexibility and maneuverability since micron-sized Ag fibers with various diameters exhibit similar scattering efficiencies in the ATSW. The presence of the metal core results in increased backscattering components compared to pure polymer fiber counterparts (Figure S7), thereby reducing the number of required scattering events for reversing incident light (Figure S8A). The unique fiber structure enables higher LWIR reflectivity compared to pure polymer strategies^48^ (Figures S8B and S8C) while maintaining breathability (Figure S9). To achieve the desired core-shell structure, we employed a modified dip-coating method (Figure 2A) (see fabrication of the fabric in method details), which allowed the application of an 800 nm-thick PE coating onto the surface of Ag fibers (Figures 2D and 2E) for protection against the oxidation of the metal core.

After completing the aforementioned process, the three functional layers of the DM fabric, with thicknesses as indicated in Table S1, were seamlessly joined together through lamination and stitching. By harnessing the knittability of fiber structures, our preparation process effortlessly adapts to industrial equipment, thereby unlocking its potential for scalable manufacturing. The spectral characteristics of the DM fabric in white color are depicted in Figure 2F. The radiative cooling side of the DM fabric exhibits a brilliant white appearance with a solar reflectance of ∼92% and an average emissivity of ∼94% within the ATSW band. And the thermal insulation side exhibits a suppressed emissivity of less than 30% in the ATSW band. The low emissivity of the thermal insulation side also helps the DM fabric minimize heat transfer from the external environment to the internal space when operating in cooling mode (Figure 1B). This remarkable optical performance is attributed to strong interactions between specific optical wavebands and scatterers composed of various sizes and materials selected during experimentation.

Coloring is also possible with the proper doping of commercially available dyes (Figure 2G). Fe_2_O_3_, Sudan Blue II, and Tartrazine (Figure S10) are doped into PMMA solutions for spraying a thin coloring coating for the cooling side (Figure S11). Additionally, by incorporating IR-transparent pigments (IRTPs) such as ZnO, Fe_2_O_3_, Prussian blue (PB), Si nanoparticles (SiNPs), and so forth (Figure S10) into the shell layer, coloring can be achieved on the thermal insulation side (Figure 2A, inset; Figures S12 and S13).

In terms of coloring on the cooling side, the large penetration depth of NIR light makes it only experience a short optical path in the colored layer before entering the UV reflective layer and the radiative cooling layer, where it undergoes intense scattering and reflection back into the environment (Figure 2H). Consequently, when compared to commercially available cotton fabrics in similar colors, such structured DM fabrics can effectively reduce near-infrared solar radiation absorption by 42% (red), 66% (blue), and 69% (yellow) respectively; furthermore, total solar radiation absorption can be reduced by 14% (red), 33% (blue), and 44% (yellow) respectively (Figure S14).

On the thermal insulation side, the incorporation of IRTPs enables fibers to exhibit different colors (Figure 2I). The nanoscale size and negligible infrared absorption of the IRTPs have minimal impact on the optical characteristics of the PE shell within the ATSW band (Figure S15), thus maintaining a low emissivity of less than 30% (Figures 2F and 2I). The significant difference in LWIR emissivity allows both sides of DM fabric to exhibit completely opposite thermal radiation characteristics under an IR camera (Figure 2G).

### Thermal management performance of the dual-mode fabric

**Figure 3:**
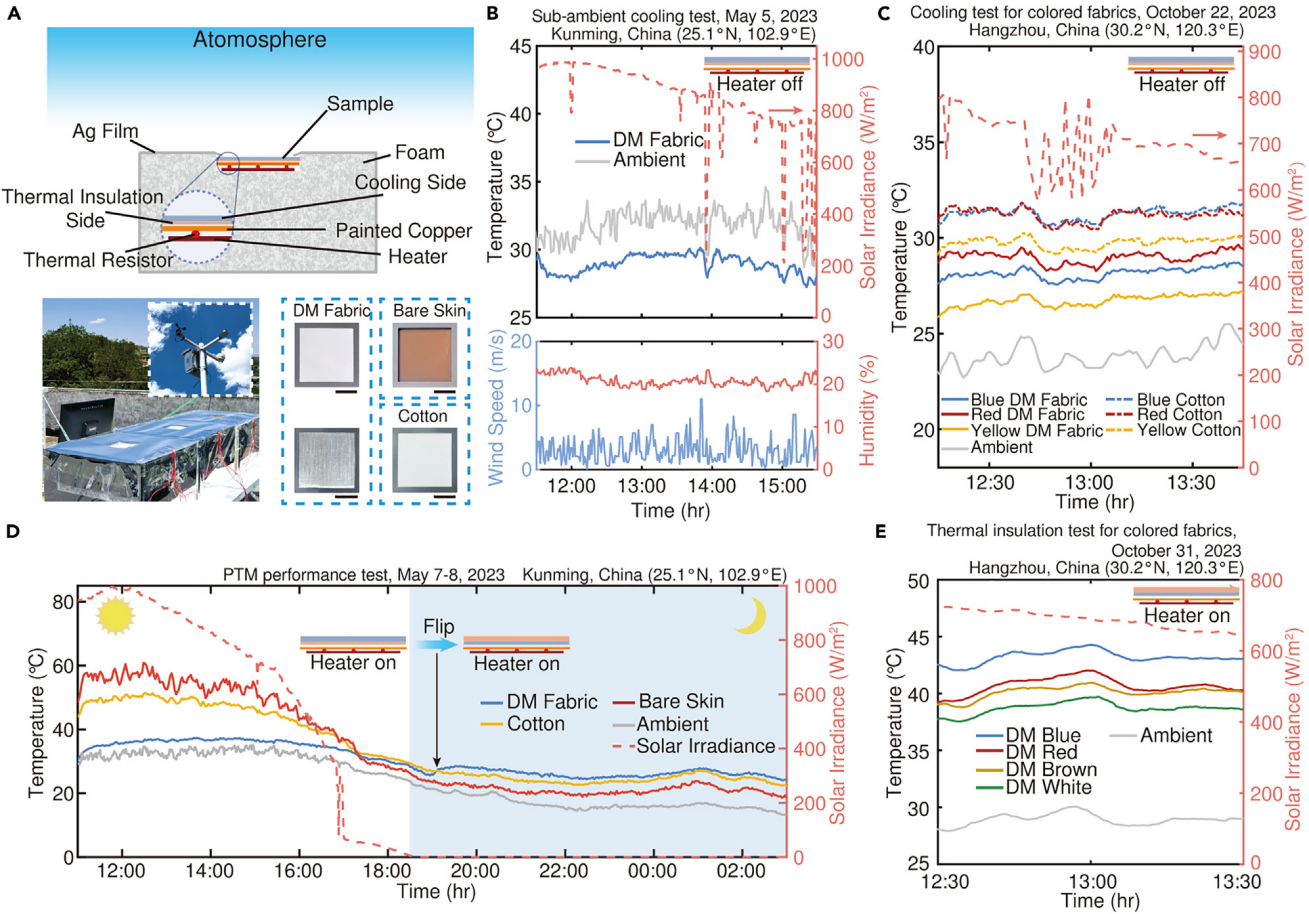
Thermal management characterization of the DM fabric (A) Schematic and photographs of the thermal measurement system and samples used to characterize the thermal management performance. Scale bars, 4 cm. (B) Temperature measurement of the sub-ambient cooling performance test. (C) Temperature measurements of the cooling effect of colored samples. (D) PTM performance test of the DM fabric using skin simulators. Reverse thermal management effects can be achieved during the day and night through flipping operations. (E) Daytime temperature measurement of the DM fabrics in thermal insulation mode. Absorption of solar radiation leads to a further rise in skin simulators’ temperature.

The thermal management performance of the DM fabric was evaluated using the devices depicted in Figure 3A (see thermal measurement devices of the fabrics in method details). The sample was placed on a thin painted copper plate that simulated human skin. Temperature measurement was achieved by incorporating thermal resistors beneath the copper plates, preventing any errors resulting from sunlight absorption. The bottom heater was used to mimic the metabolic heat generated by the human body. In order to simulate a more realistic application scenario for the DM fabric, we intentionally omitted the integration of PE film on the device, which is typically used as insulation against factors such as heat convection. Initially focusing on assessing its daytime radiative cooling capability, we deactivated the heater and positioned the white DM fabric with the cooling side facing upwards. Under the solar power of ∼1000 W/m^2^ at noon time (11:45 - 12:15), the DM fabric achieved a daytime radiative cooling effect of ∼2.9°C lower than the ambient temperature (Figure 3B). Despite some solar energy absorption due to its coloring, thanks to higher reflection in NIR band wavelengths, colored variants of DM fabrics exhibited lower temperatures compared to commercially available products in similar colors. Specifically, blue-, red-, and yellow-colored DM fabrics demonstrated average temperature reductions of 3.1°C, 2.5°C, and 3.0°C respectively (Figure 3C).

In the simulated PTM test (Figure 3D), upon activating the heater, the DM fabric effectively maintained the temperature of the skin simulator below 37.3°C during the daytime. This represents a remarkable temperature reduction exceeding 24°C and 15°C compared to bare skin and cotton fabric covered skin, respectively. Following sunset (18:30), we flipped the DM fabric, whereby its outer surface with low LWIR emissivity impeded heat dissipation, resulting in an elevation of temperatures by over 6°C and 3°C compared to bare skin and cotton fabric correspondingly. It is noteworthy that coloring on the thermal insulation side enhances solar radiation absorption, thereby augmenting the thermal management performance of the DM fabric to some extent. As shown in Figure 3E, due to increased solar radiation absorption by around 28% and 40% relative to white coloration, the DM fabrics with brown and red colors were able to raise temperatures of skin simulators by approximately 1.4°C and 2.0°C. Importantly, PB exhibits absorption extending into wavelengths as deep as 1 μm (Figure 2E), enabling it to absorb more solar radiation than other colors, consequently leading to a temperature increase of approximately 4.5°C for covered skin simulator compared to that covered with white fabric.

**Figure 4:**
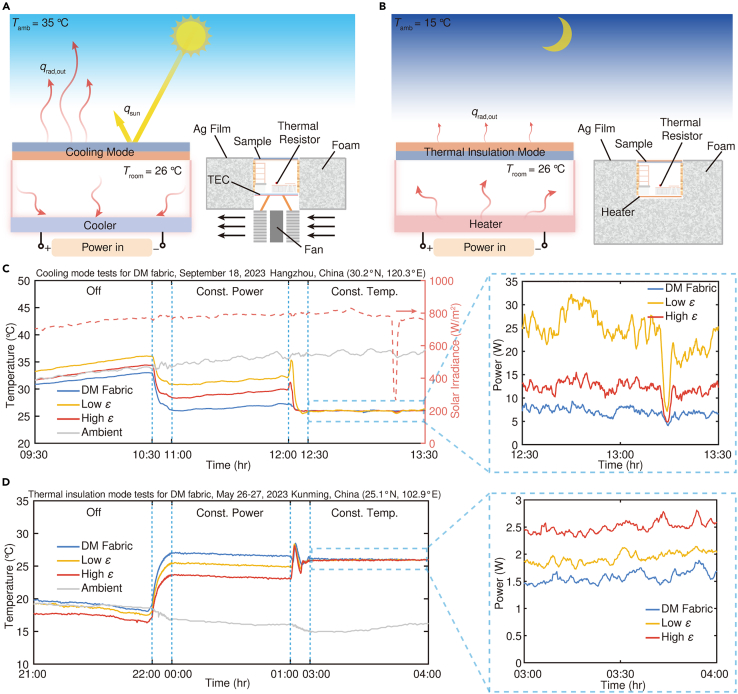
Application of the DM fabric as the canopies for ITM (A) Schematics of the thermal measurement system used to characterize the cooling performance for the indoor environment. (B) Schematics of the thermal measurement system used to characterize the heating performance for indoor environment. (C) Continuous temperature measurement of the indoor environment under different working conditions of TECs, in cooling mode during the daytime. The inset shows the power of cooling devices using different samples during the constant temperature stage. (D) Continuous temperature measurement of the indoor environment under different working conditions of heaters, in thermal insulation mode during the nighttime. The inset shows the power of heating devices using different samples during the constant temperature stage. Low *ε*/high *ε*, commercial shade cloths with low/high LWIR emissivity.

The exceptional flexibility, customizability, and seamless integration of the DM fabric render it an exemplary thermal management platform in the form of architextiles for a diverse range of ITM scenarios encompassing buildings, vehicles, and tents. In order to authenticate its efficacy in indoor environments with regards to thermal regulation and energy conservation, we conducted experiments (Figures 4A and 4B) (see thermal measurement devices of the fabrics in method details) employing miniature house models (Figure S16). Canopies, made with white DM fabric as well as commercially available shade cloths with varying spectral properties (Figure S17) were placed atop these models.

During the daytime, the cooling side of the DM fabric faced outward (Figure 4A). The asymmetric spectral design allows it to reflect a majority of the solar radiation, enhance its thermal radiation to the sky, and simultaneously minimize its own thermal radiation to the internal environment. As shown in Figure 4C, during the initial two stages with no external cooling source or Thermoelectric Coolers (TECs) operating at constant power, the model covered with the DM fabric exhibited the lowest indoor temperature due to its exceptional radiative cooling performance. In the next stage where a constant temperature is maintained, an activated temperature control system regulates the input power of TECs. Remarkably, compared to the other two commercial shade cloths, only minimal cooling power was required by the model covered with the DM fabric to maintain a comfortable indoor temperature of 26°C. The results unequivocally indicate that the utilization of DM fabric resulted in energy savings of 70% (Low *ε*) and 42% (High *ε*), respectively.

During cold nighttime when sunlight is not available and heating demand is at its peak, the DM fabric was flipped over to activate thermal insulation mode (Figure 4B). The TECs were replaced with heaters to simulate the heating mode of HVAC systems. As shown in Figure 4D, owing to the outer surface’s lowest emissivity, the model covered with the DM fabric maintained the highest indoor temperature during the first two stages. Similar to setting the indoor temperature of the three models at 26°C for cooling mode, the DM-fabric-covered model could achieve approximately 19% (Low *ε*) and 37% (High *ε*) savings in heating energy consumption, respectively.

## Discussion

### Energy saving potential

**Figure 5:**
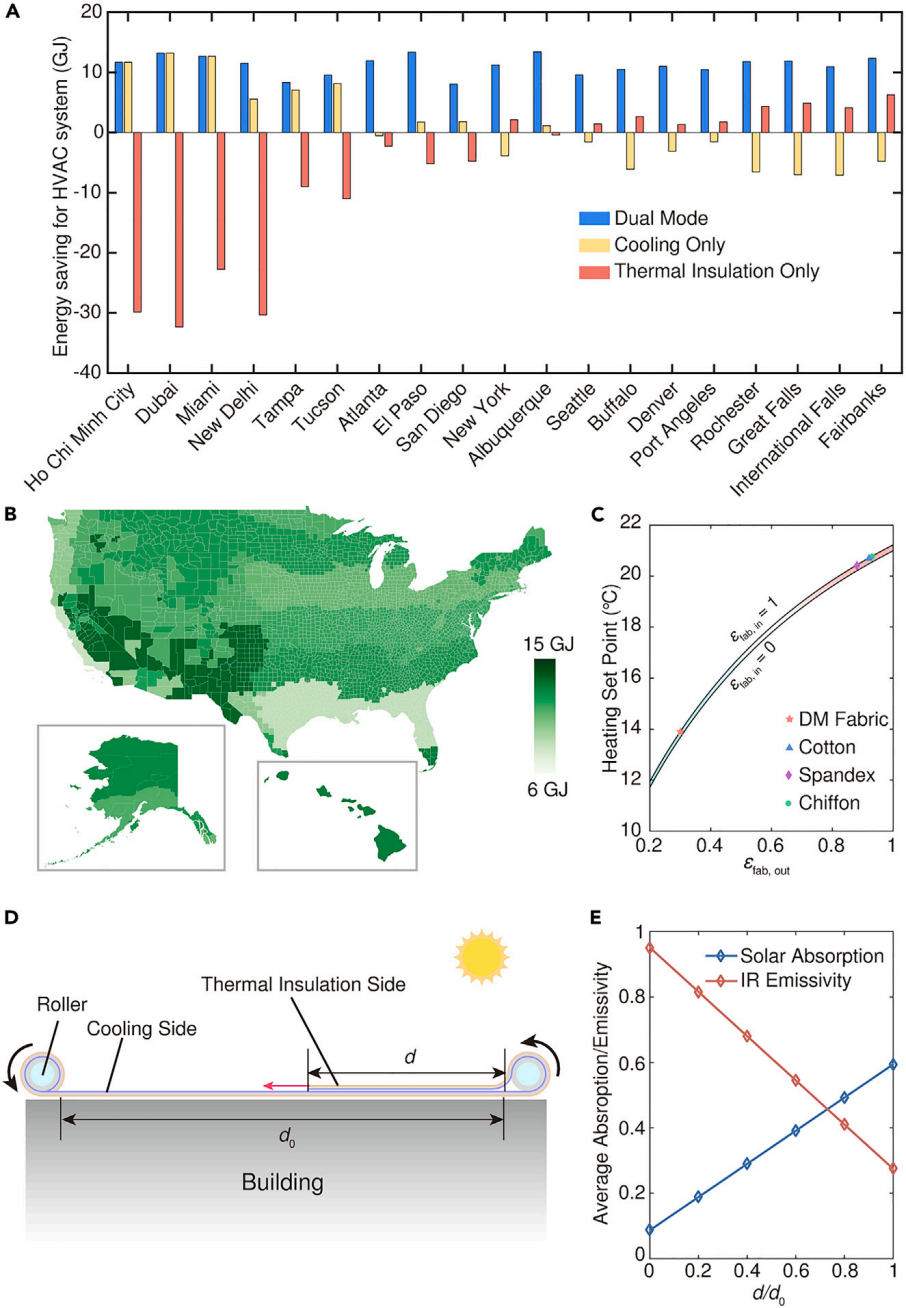
Assessment of energy saving potential (A) Calculated annual energy saving data on HVAC systems of buildings in representative cities after applying the DM fabric, cooling fabric, and thermal insulation fabric to the roofs. (B) Annual energy consumption reductions for HVAC systems of midrise apartment buildings in US cities after applying the DM fabric to the roofs. (C) Heating set points for fabrics with different surface emissivity when used for indoor PTM. *ε*_fab,in_/*ε*_fab,out_, inner/outer surface emissivity of the fabric. (D) Schematic diagram of a strategy to achieve continuous dynamic thermal management based on the DM fabric with mechanical assistance. (E) Continuous absorption/emissivity regulation achieved by the device under different working conditions (*d*/*d*_0_).

It is profitable to consider the potential of dual-mode radiative thermal management in energy savings. Currently, this approach presents a realistic and feasible solution due to its straightforward principle, low manufacturing complexity, high operability and commendable thermal management performance. To evaluate the energy-saving capabilities of the DM fabric, we conducted an energy consumption simulation using a typical 4-story mid-rise apartment model (calculating of the energy consumption in method details). By applying the DM fabric on the roof to serve as architextiles and adjusting its thermal management mode monthly based on specific climate conditions, cities in different climate zones will have customized operating schedules of the DM fabrics to optimally adapt to the environment of the region (Figure S18). As a result, we observed significant positive energy-saving effects across representative cities in all climate zones (Figures 5A and 5B). These effects translate into annual energy savings ranging from 8 GJ (∼2200 kWh) to 14 GJ (∼3900 kWh), surpassing the limitations of fixed optical properties. One remarkable advantage of DM fabrics lies in their versatility as they can also be utilized for curtains, awnings, and other products to further reduce HVAC system energy consumption. The DM fabric’s thermal insulation mode also proves valuable for indoor PTM. Calculation results based on a steady-state heat transfer model (Note S1) demonstrate that wearing the DM fabric can lower the temperature set point for maintaining human thermal comfort by over 6.5°C compared to various fabrics with similar thicknesses (Figure 5C). This signifies a substantial impact on reducing the energy consumption of heating systems. Additionally, it is worth mentioning that with the assistance of some mechanical structures, the DM fabric can easily transition from a dual-mode thermal management device to a continuous dynamic thermal management device. As shown in Figure 5D, the reasonable cooperation of the two rollers can control the ratio of the cooling and thermal insulation sides of the DM fabric, thereby achieving continuous regulation of its radiation properties (Figure 5E).

### Conclusion

The DM fabric proposed in this study offers a scalable solution for both PTM and ITM applications covering most scenarios encountered in daily thermal management. With its multilayered configuration and hierarchical micro-nano structures, the DM fabric achieves selective spectrum control spanning from 0.3 to 20 μm, enabling the regulation of optical, thermal, and aesthetic properties such as solar reflectivity, infrared emissivity, and coloring. Through an asymmetric spectrum design, the DM fabric effortlessly transitions between radiative cooling and thermal insulation modes as required. Experimental demonstrations and energy-saving simulations have unequivocally demonstrated that the DM fabric outperforms commercial fabrics in terms of thermal control capabilities. Compared to cotton, the Janus fabric can decrease/increase skin simulator temperature by over 15°C/3°C during daytime/nighttime respectively. In the ITM test, when using the DM fabric as the shade cloth, the consumed power of the heating/cooling system could be significantly reduced compared with commercial products. In cooling mode, the utilization of DM fabric could result in energy savings of 70% (Low *ε*) and 42% (High *ε*), respectively. While in heating mode, it would achieve approximately 19% (Low *ε*) and 37% (High *ε*) savings in heating energy consumption, respectively. Covering building roofs with fabric possessing such excellent spectral properties is also predicted to achieve year-round positive energy savings for HVAC systems in all climate types. Through intelligent combination with some mechanical structures, the DM fabric is expected to achieve continuously adjustable customized dynamic thermal management, which is necessary for a device that meets actual needs and still faces considerable challenges for pure material innovation. Consequently, it holds immense potential for diverse applications ranging from PTM cases (clothing and outdoor protection) to ITM cases (architextiles for buildings, vehicles, and home furnishings). These applications are pivotal in addressing real-life challenges faced by humanity today. By acknowledging the potential of dual-mode radiative thermal management, we can drive energy efficiency forward while paving the way for sustainable developments. Further enhancement of thermal regulation efficiency and reduction of manufacturing costs could be achieved through meticulous material optimization and process improvement endeavors. For instance, replacing the Ag core of LWIR shielding fibers with more cost-effective alternatives such as stainless-steel filament is one avenue worth exploring. Additionally, delving into advanced materials such as photoluminescent materials^43^ and MXene fibers^49^^,^^50^ may expand the spectral response range of the DM fabric while optimizing its absorption capacity for solar power.

### Limitations of the study

Further research is needed to refine the spectrum. In the case of coloring, the absorption of the UV band by the cooling side will increase the heat input and will not have a beneficial effect on coloring. The utilization (absorption) of LWIR shielding fibers for solar radiation in the NIR band is still limited. Since it is to be worn on both sides, it is necessary to further explore the hydrophilic structure of the front and back of the fabric to achieve symmetrical wearing comfort in the case of an asymmetric spectrum. In addition, the efficiency of radiative thermal management is limited by humidity and cloudiness. In the case of high humidity or high cloudiness, the atmospheric transmittance will be significantly reduced, thus reducing the performance of radiative thermal management.

## Resource availability

### Lead contact

Further information and requests for resources should be directed to and will be fulfilled by the lead contact, Yaoguang Ma (mayaoguang@zju.edu.cn).

### Materials availability

This study did not generate new materials.

### Data and code availability

• All data reported in this article will be shared by the lead contact upon request.
• No original code was used.
• Any additional information required to reanalyze the data reported in this article is available from the lead contact upon request.

## Acknowledgments

This work is supported by the 10.13039/501100001809National Natural Science Foundation of China (NSFC) grant (62222511 (Y. M.) and 61905213 (Y. M.)), 10.13039/501100012166National Key Research and Development Program of China grant (2023YFF0613000 (Y. M.)), 10.13039/501100004731Natural Science Foundation of Zhejiang Province China grant (LR22F050006 (Y. M.)) and the STI 2030–Major Projects grant (2021ZD0200401 (Y. M.)). The author would like to acknowledge Weige Lv, Liying Chen, and Wei Wang from the State Key Laboratory for Extreme Photonics and Instrumentation and College of Optical Science and Engineering, Zhejiang University for their assistance in experiments.

## Author contributions

Y.M. and S.P. conceptualized the idea and established the methodology. S.P. conducted modeling and simulation. S.P. and Z.W. performed experiments and analyzed the data. Y.M. and S.P. prepared the initial draft. All authors contributed to the discussion of results, article review, and editing. Y.M supervised the research project.

## Declaration of interests

The authors declare no competing interests.

## STAR★Methods

### Key resources table

**Table:** 

REAGENT or RESOURCE	SOURCE	IDENTIFIER
**Chemicals, peptides, and recombinant proteins**		

TiO_2_	Venator	ALTIRIS 550
PLA	NatureWorks	6100D; CAS: 26100-51-6
PTFE	NIVO New Material Co., Ltd	N/A
LDPE	Usolf	2426H; CAS: 9002-88-4
PMMA	Sinopharm	M.W. 35000; CAS: 9011-14-7
Acetone	Sinopharm	CAS: 67-64-1
Toluene	Sinopharm	CAS: 108-88-3

**Software and algorithms**		

MATLAB	MathWorks	https://www.mathworks.com
EnergyPlus	U.S. Department of Energy’s Building Technologies Office	https://energyplus.net

**Other**		

Spectrophotometer	Agilent	Cary 5000 UV-Vis-NIR
Fourier transform infrared spectrometer	Bruker	INVENIO R
IR camera	Fluke	Ti400
Scanning Electron Microscope	Zeiss	Utral 55

### Method details

#### Materials

The raw materials for the preparation of the fabric samples include TiO_2_ particles (Venator, ALTIRIS550), PLA (NatureWorks), PTFE (NIVO New Material), Ag filament (Yueqing Zhongjin Metal), LDPE (Usolf) and PMMA (Sinopharm). Fe_2_O_3_ (Macklin, α-Fe_2_O_3_, 30 nm), Sudan Blue II (Aladdin), Tartrazine (Rhawn), ZnO (Bocheng Metallurgical, 500 nm), Prussian blue (Macklin), Si nanoparticles (Macklin, crystalline, 100 nm) were used as colorants for the DM fabrics. Acrylic ester aqueous polymer (Wujiang Tianli Polymer, WJ-68) was used as the sizing agent for weaving. Acetone (purity ≥99.5%) was used to dissolve PMMA for coating. Toluene (purity ≥99.5%) was used to dissolve PE for coating. All materials were used as supplied.

#### Fabrication of the fabric

The radiative cooling layer woven by TiO_2_-PLA metafibers acts as a support for the entire fabric. The preparation process followed a similar procedure outlined in the Zeng et al.^19^ Initially, TiO_2_ particles were mixed with PLA at a volume ratio of TiO_2_: PLA = 3:17, at a temperature of 205°C, using a twin-screw extruder. The resulting mixture was then cut into composite master batches with a pelletizer operating at 300 rpm. The TiO_2_-PLA composite master batches were dried for 24 h at 130°C. Next, the metafibers were spun from the dried composite master batches using a standard double-screw melt-spinning machine, set at 205°C. By employing the drafting process on a draw winder machine, metafibers with a diameter of approximately 30 μm were obtained. These metafibers were then combined on an air covering machine, where two 240 Denier (D)/24 Filaments (F) were twisted together to form plied yarns using a two-for-one twister. To release internal stress, enhance strength, and improve ductility, the yarns were steamed in a common steam box at 100°C and 80% humidity for 30 min. Following this, yarn sizing (GA392, Jiangyin Tongyuan Textile Machinery), warping (GA193, Jiangyin Tongyuan Textile Machinery), and weaving processes (SGA598, Jiangyin Tongyuan Textile Machinery) were carried out to form the fabric. The radiative cooling layer was then thoroughly rinsed with deionized water (DIW) at 40°C to remove the sizing agent.

LWIR shielding fibers with core-shell structure were prepared by dip-coating method.^51^ First, we take IRTPs, LDPE and toluene with a volume ratio of 1:9:110. The IRTPs were mixed with toluene and dispersed ultrasonically at 600 W for 2 h. LDPE was then added to the dispersion and heated with stirring at 85°C until it was completely dissolved and then maintained at the same temperature. Subsequently, using homemade dip-coating equipment, the surfaces of the Ag filaments were coated with the toluene solution of IRTPs-LDPE and the toluene was quickly evaporated by a 120°C heater when pulled out (Figure 2A). The coating speed was set to ∼360 m/h, and the coating efficiency could be improved through multi-filament parallel coating process.

Then, by closely arranging the warp and weft of the LWIR shielding fibers and stitching them on the radiative cooling layer to get the non-crimp fabric layer, and laminating an 80 μm-thick PTFE clothing film on the other side, we obtained the DM fabric with a thickness of ∼630 μm.

For the coloring of the cooling side, first we configured the acetone dispersions of Sudan Blue II, Fe_2_O_3_, and Tartrazine with the concentration of 2 mg/mL, 50 mg/mL and 2 mg/mL, respectively. After mixing evenly, the colored PMMA-acetone-water precursor solution was made with the mass ratio of 1:8:1. The solution with different color was then spray-coated on the cooling side of the DM fabric.

#### Optical characterization

A spectrophotometer (Agilent, Cary 5000 UV-Vis-NIR) with an integrating sphere (Agilent, Internal DRA2500) was used to measure reflectivity and transmissivity spectra in the 0.3–2.5 μm range. A Fourier transform infrared spectrometer (Bruker, INVENIO R) equipped with a gold integrating sphere (Bruker, A562) was used to measure the IR emissivity spectra in the 2.5–20 μm range. An IR camera (Fluke Ti400) was used to take thermal images of the samples.

#### Water vapor transmission test

The water vapor transmission rate of fabric samples was tested according to GB/T 12704.2-2009: The permeability cups were filled with 34 mL distilled water and sealed by the samples using silicone gaskets to form the test ensembles. The exposed area of fabrics is 6 cm in diameter. The test ensembles were then placed into an environmental chamber. The temperature was maintained at 38°C and relative humidity at 50%. After 1 h balance, the mass of the test ensembles was then measured periodically, and the reduced mass should come from the evaporated water. The reduced mass was then divided by the area and test time to derive the water vapor transmission.

#### Thermal measurement devices of the fabrics

The fabric samples were carefully positioned inside a foam container, raised 0.7 m above the ground to minimize heat transfer and prevent significant heat loss. The thermal measurement apparatus was covered with a layer of silver film, which effectively reflected both solar power and thermal radiation from the surroundings. Each sample was attached to a painted copper plate, with a Kapton heater positioned beneath each plate. The samples’ surfaces faced the sky through square windows with 10 cm side length. The ambient temperature, solar radiation power, wind speed and humidity were collected using commercial meteorological terminals. For the skin simulators test, the heaters were switched on with a power density of 101 W/m^2^.

For testing the ITM performance of the samples, a square pit was dug out in the center of each foam container. A miniature house model (12.7 cm × 10 cm × 10 cm) was placed within the pit with a TEC/heater placed underneath it for cooling/heating needs. Fabric samples were draped on top of the models to serve as their canopies. A small fan was placed inside each model to increase convection and make the temperature distribution relatively even. Thermal resistors were placed in exactly the same location to ensure consistency in temperature measurements across different models. A homemade PID control system was used to control the temperatures inside the models and record the energy consumption of the HVAC systems (TECs/heaters and fans).

#### Heat transfer analysis of the structure

A steady-state heat transfer model analysis was used to evaluate the heat flux of the fabric to the indoor environment and calculate the heating set point when applied to indoor PTM.

When calculating the net heat flux to the indoor environment of fabric as canopy (Figure 1B), assuming that the room can be regarded as a black body with constant temperature, the energy balance equations of the inner and outer surfaces of the fabric are (Figure S19A).

(Equation 1) $$q_{\text{rad},\text{out}}+q_{\text{conv},\text{out}}+q_{\text{cond}}=q_{\text{solar}}+q_{\text{atm}}$$

(Equation 2) $$q_{\text{rad},\text{in}}+q_{\text{conv},\text{in}}=\varepsilon_{\text{in}}\cdot q_{\text{rad},\text{room}}+q_{\text{cond}}$$

where

(Equation 3) $$q_{\text{solar}}=\int d\lambda \cdot I_{\text{solar}}\left(\lambda \right)\cdot \alpha_{\text{out}}\left(\lambda \right)$$

(Equation 4) $$q_{\text{atm}}=\int_{0}^{\frac{\pi }{2}}d\theta \cdot \pi \;\sin\left(2\theta \right)\int d\lambda \cdot I_{\text{BB}}\left(T_{\text{amb}},\lambda \right)\varepsilon_{\text{out}}\left(\lambda \right)\left({1-\tau_{\text{atm}}\left(\lambda \right)}^{1/\cos\left(\theta \right)}\right)$$

(Equation 5) $$q_{\text{rad},\text{out}}=\varepsilon_{\text{out}}\sigma T_{\text{out}}^{4}$$

(Equation 6) $$q_{\text{rad},\text{in}}=\varepsilon_{\text{in}}\sigma T_{\text{in}}^{4}$$

(Equation 7) $$q_{\text{rad},\text{room}}=\sigma T_{\text{room}}^{4}$$

(Equation 8) $$q_{\text{cond}}=k_{\text{fab}}\cdot \frac{T_{\text{out}}-T_{\text{in}}}{t_{\text{fab}}}$$

(Equation 9) $$q_{\text{conv},\text{out}}=h_{\text{out}}\cdot \left(T_{\text{out}}-T_{\text{amb}}\right)$$

(Equation 10) $$q_{\text{conv},\text{in}}=h_{\text{in}}\cdot \left(T_{\text{in}}-T_{\text{room}}\right)$$

And the heat flux of fabric to the indoor environment could be calculated

(Equation 11) $$q_{\text{net},\text{in}}=q_{\text{rad},\text{in}}+q_{\text{conv},\text{in}}-\varepsilon_{\text{in}}\cdot q_{\text{rad},\text{room}}$$

based on the values of parameters listed in Table S2.

When calculating the effect of fabrics on heating set points in indoor PTM, ignoring the convection of the air gap between the human body and the fabric and the absorption of indoor scattered sunlight, we get (Figure S19B).

(Equation 12) $${\text{Fabric}\;\text{outer}\;\text{surface}\;q}_{\text{rad},\text{out}}+q_{\text{conv},\text{out}}=\varepsilon_{\text{out}}\cdot q_{\text{rad},\text{room}}+q_{\text{cond},\text{fab}}$$

(Equation 13) $${\text{Fabric}\;\text{inner}\;\text{surface}\;q}_{\text{rad},\text{in}}+q_{\text{cond},\text{fab}}=\varepsilon_{\text{in}}\cdot q_{\text{rad},\text{skin}}+q_{\text{cond},\text{skin}}$$

(Equation 14) $${\text{Skin}\;\text{surface}\;q}_{\text{gen}}+q_{\text{rad},\text{in}}=\varepsilon_{\text{in}}\cdot q_{\text{rad},\text{skin}}+q_{\text{cond},\text{skin}}$$

where

(Equation 15) $$q_{\text{rad},\text{out}}=\varepsilon_{\text{out}}\sigma T_{\text{out}}^{4}$$

(Equation 16) $$q_{\text{rad},\text{in}}=\varepsilon_{\text{in}}\sigma T_{\text{in}}^{4}$$

(Equation 17) $$q_{\text{rad},\text{room}}=\sigma T_{\text{room}}^{4}$$

(Equation 18) $$q_{\text{rad},\text{skin}}=\sigma T_{skin}^{4}$$

(Equation 19) $$q_{\text{cond},\text{fab}}=k_{\text{fab}}\cdot \frac{T_{\text{in}}-T_{\text{out}}}{t_{\text{fab}}}$$

(Equation 20) $$q_{\text{cond},\text{fab}}=k_{\text{fab}}\cdot \frac{T_{\text{in}}-T_{\text{out}}}{t_{\text{fab}}}$$

(Equation 21) $$q_{\text{conv},\text{out}}=h_{\text{out}}\cdot \left(T_{\text{out}}-T_{\text{room}}\right)$$

And the heating set points to maintain human body at 34°C could be calculated based on the values of parameters listed in Table S3.

#### Scattering properties of particles or fibers

The scattering and absorption properties of single particles were approximated using spheres based on the Lorenz-Mie theory.^52^ For the case of doping particles into polymers, far-field approximation (FFA)^53^ was introduced to take into account the absorption of the matrix material. Considering that the refractive index of the matrix and particles is $m_{0}=n+ik$ and $m_{1}=n^{\prime }+ik^{\prime }$, respectively. The scattering efficiency $Q_{S,p}$ and extinction efficiency $Q_{e,p}$ can be calculated by

(Equation 22) $$Q_{S,p}=C_{\text{FF}}\sum_{n=1}^{\infty }\left(2n+1\right)\left({\left|a_{n}\right|}^{2}+{\left|b_{n}\right|}^{2}\right)$$

(Equation 23) $$Q_{e,p}=C_{\text{FF}}\sum_{n=1}^{\infty }\left(2n+1\right)\text{Re}\left(a_{n}+b_{n}\right)$$

The FFA correction factor $C_{\text{FF}}$ in can be expressed as

(Equation 24) $$C_{\text{FF}}=\frac{4k^{2}\;\exp\left(-2kx\right)}{\left(n^{2}+k^{2}\right)\left[1+\left(2kx-1\right)\exp\left(2kx\right)\right]}$$

where x=2πR/λ denotes the size parameters of particles, R is the radius of particles, and λ is the wavelength of electromagnetic waves. The expressions of Mie scattering coefficients $a_{n}$ and $b_{n}$ can be found in Yin and Pilon.^53^

The scattering and absorption properties of a fiber can be calculated by treating it as an infinitely long concentric cylinder. According to the polarization state of the incident light, for the case where the electric field direction is parallel to the fiber

(Equation 25) $$Q_{S,f}=\frac{2}{x}\sum_{n=-\infty }^{\infty }{\left|b_{n}\right|}^{2}$$

(Equation 26) $$Q_{e,f}=\frac{2}{x}\sum_{n=-\infty }^{\infty }\text{Re}\left(b_{n}\right)$$

For the case where the electric field direction is perpendicular to the fiber

(Equation 27) $$Q_{S,f}=\frac{2}{x}\sum_{n=-\infty }^{\infty }{\left|a_{n}\right|}^{2}$$

(Equation 28) $$Q_{e,f}=\frac{2}{x}\sum_{n=-\infty }^{\infty }\text{Re}\left(a_{n}\right)$$

And the asymmetric parameter

(Equation 29) $$g=\left\langle \cos\;\theta \right\rangle =\int_{0}^{2\pi }\frac{\lambda {\left|T\left(\theta \right)\right|}^{2}}{2\pi^{2}RQ_{S\_f}}\cos\;\theta d\theta$$

Here, R is the radius of the whole fiber, and T(θ) is the amplitude function. The expressions of scattering coefficients $a_{n}$ and $b_{n}$ can be found in Kerker and Matijević.^54^ The final scattering and absorption efficiency could be given by the average of the two cases.

#### Calculating of fabric emissivity

To calculate the emissivity in the LWIR band, effective medium theory (EMT)^55^^,^^56^ and finite-difference time-domain (FDTD) numerical simulations were used to construct an equivalent model of the fabrics. Based on EMT, we consider the polymer matrices doped with nanoparticles (IRTPs, TiO_2_, etc.) as homogeneous media. Assuming that the particles are distributed in a single size, if the number of particles per unit volume is N, the effective parameters $\varepsilon_{\text{eff}}$ and $\mu_{\text{eff}}$ are given by

(Equation 30) $$\frac{\varepsilon_{\text{eff}}-\varepsilon_{m}}{\varepsilon_{\text{eff}}+2\varepsilon_{m}}=\frac{2\pi iN}{k_{m}^{3}}a_{1}$$

(Equation 31) $$\frac{\mu_{\text{eff}}-1}{\mu_{\text{eff}}+2}=\frac{2\pi iN}{k_{m}^{3}}b_{1}$$

where $\varepsilon_{m}$ is the permittivity of the matrix, $k_{m}$ is the wave vector in the matrix and $\mu_{0}$ is the permeability of the vacuum. Thus, the effective refractive index is $n_{\text{eff}}=\sqrt{\mu_{\text{eff}}\varepsilon_{\text{eff}}}$. Based on the calculated effective refractive index, fibers can be simplified into uniform structures (TiO_2_-PLA metafibers) and uniform core-shell structures (LWIR shielding fibers). Then, simple 2D FDTD models with periodic boundary conditions were constructed to calculate the LWIR emissivity of the fabrics, by randomly distributing these equivalent fibers in the space. For radiative cooling layer/LWIR shielding non-crimp fabric layer, the thickness of the fabric was set to 400 μm/150 μm, and the volume fraction was set to 35%/40%, based on parameters obtained in experiments.

#### Calculating of the energy consumption

EnergyPlus (version 22.1) was used to calculate the energy consumption of a typical 4-story mid-rise apartment building.^57^ We treated the reference building model as the baseline, and then applied the DM fabric on the roof of the model to determine the energy saving performances. The DM fabric was set to be white color in cooling mode and blue color in thermal insulation mode, to maximize the passive cooling and heating power, respectively. The outer surface reflectivity and emissivity data of the DM fabric in the two modes were set based on the experimentally measured spectra. On a monthly basis, the DM fabric determined its thermal management mode based on the weather conditions (Figure S17). Typical locations of all climate zones were chosen to demonstrate the application potential of DM fabric: Ho Chi Minh City (0A), Dubai (0B), Miami (1A), New Delhi (1B), Tampa (2A), Tucson (2B), Atlanta (3A), El Paso (3B), San Diego (3C), New York (4A), Albuquerque (4B), Seattle (4C), Buffalo (5A), Denver (5B), Port Angeles (5C), Rochester (6A), Great Falls (6B), International Falls (7), and Fairbanks (8).
