# Supplementary material for: Scalable colored Janus fabric scheme for dynamic thermal management
Source: iScience. 2024 Sep 13;27(10):110948. doi: 10.1016/j.isci.2024.110948 (PMC11471193; doi:10.1016/j.isci.2024.110948)
Supplement: Document S1. Figures S1–S19 and Tables S1–S3 [file mmc1.pdf]

iScience, Volume *27*

Supplemental information

Scalable colored Janus fabric scheme

for dynamic thermal management

Sijie Pian, Zhuning Wang, Chengtao Lu, Peixuan Wu, Qikai Chen, Xu Liu, and Yaoguang Ma

Supporting Figures

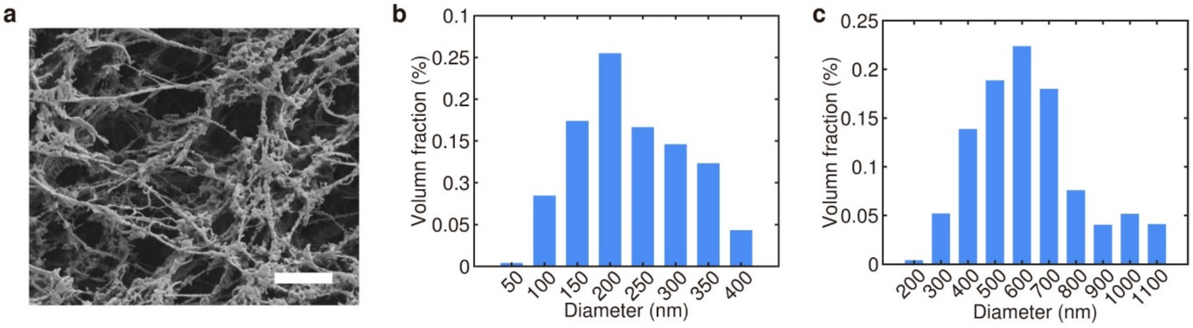

Figure S1. Morphology of the UV reflective layer (PTFE clothing film), related to Figure 2. (a) SEM image of the PTFE clothing film. Scale bar, 2 μm. (b) Size distributions of PTFE nanofibers. c, Size distributions of PTFE nanoparticles.

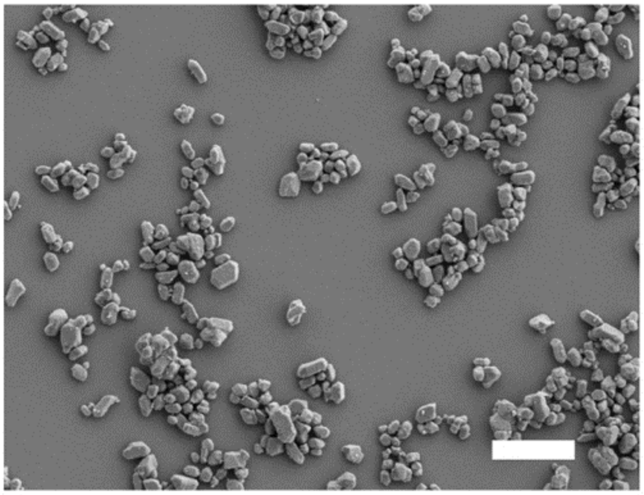

Figure S2. SEM image of the TiO_2 nanoparticles, which can strongly scatter sunlight, related to Figure 2. Scale bar, 4 μm.

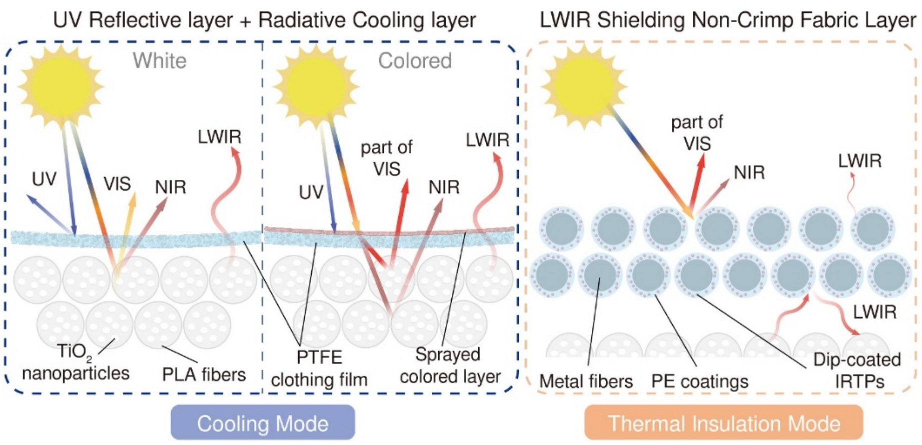

Figure S3. Structure and principles of the DM fabric, related to Figure 2.

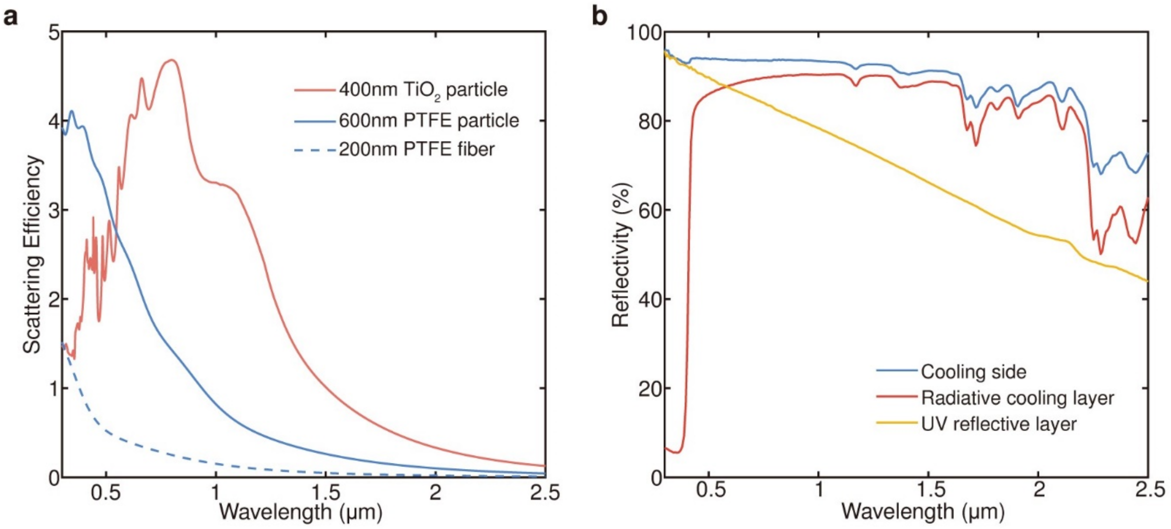

Figure S4. Optical properties of the DM fabric cooling side, related to Figure 2 and STAR methods. (a) Calculated scattering efficiency of the scatterers included in the cooling side of the DM fabric. (b) Measured overall reflectivity of the cooling side and the reflectivity of the two included structures. The scatterers of PTFE and TiO_2 have complementary scattering efficiencies, achieving broadband reflection across 0.3-2.5 μm.

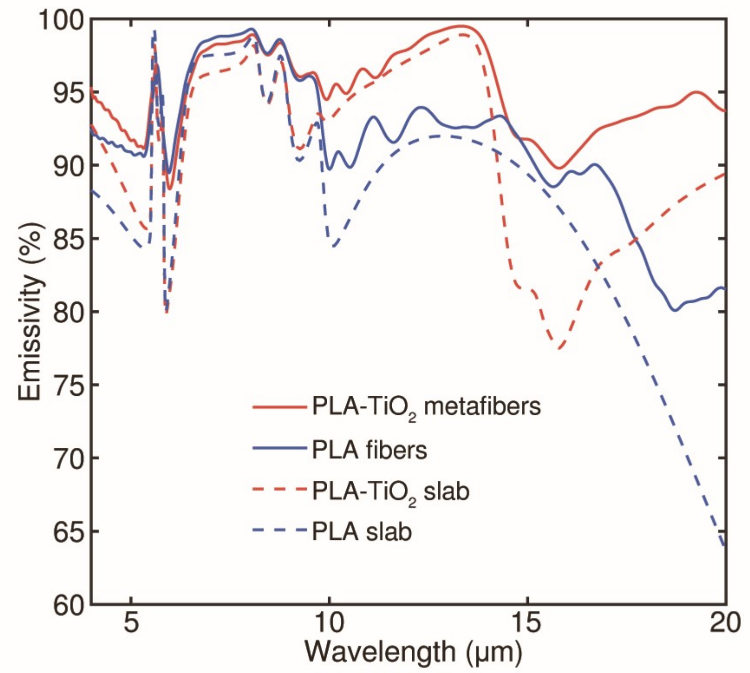

Figure S5. Calculated infrared emissivity of different structures based on FDTD simulation, related to Figure 2 and STAR methods. For the same material volume, the TiO_2-PLA metafibers have the highest emissivity due to the broadband absorption of the material, optical path increase caused by appropriate scattering intensity, and the gradient refractive index anti-reflection effect.

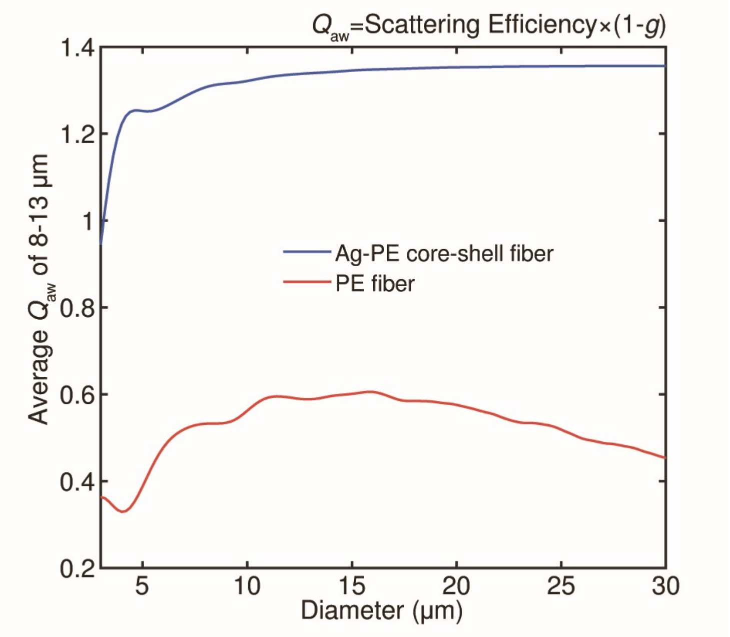

Figure S6. Calculated average angle-weighted scattering efficiency of Ag-PE core-shell fiber (LWIR shielding fiber) and pure PE fiber with different diameters in ATSW (8-13 μm), related to Figure 2 and STAR methods. The thickness of the PE-shell of the core-shell fiber is set to 800 nm based on experimental results. The results show that the scattering efficiency of Ag-PE core-shell fiber is almost unchanged when the diameter is greater than 10 μm, while the PE fiber reaches the maximum value when the diameter is in the range of 11-16 μm.

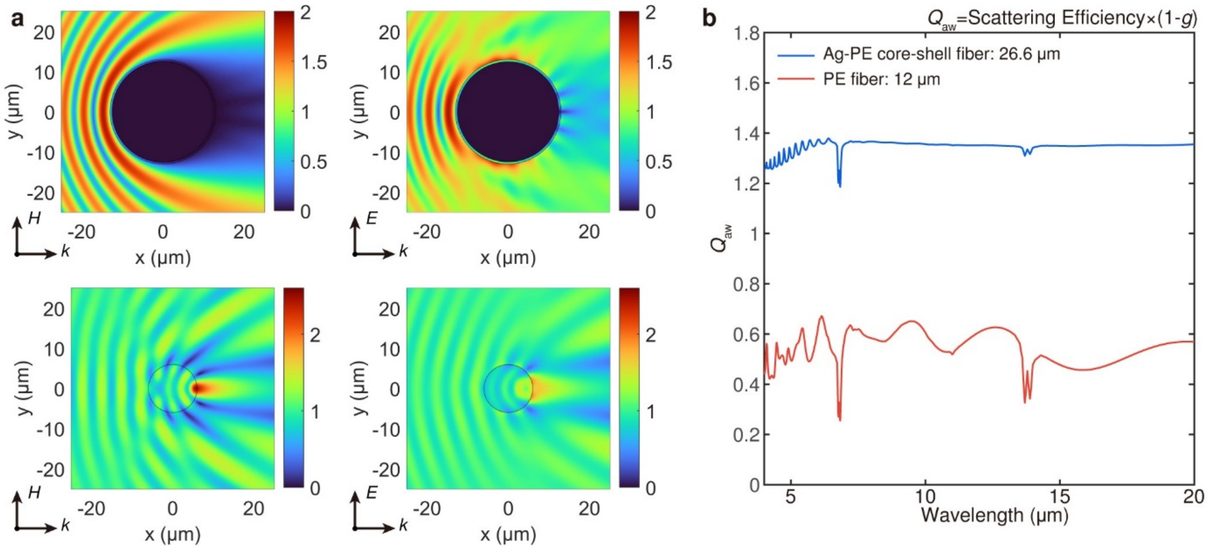

Figure S7. Calculated infrared scattering properties of Ag-PE core-shell fiber (LWIR shielding fiber) with diameter of 26.6 μm and pure PE fiber with diameter of 12 μm, related to Figure 2 and STAR methods. (a) Distribution of the simulated electric field of Ag-PE core-shell fiber and pure PE fiber for differently polarized incident light. (b) Calculated angle-weighted scattering efficiency of Ag-PE core-shell fiber and pure PE fiber. The thickness of the PE-shell of the core-shell fiber is set to 800 nm based on experimental results. The Ag-PE core-shell fiber has stronger backscattering.

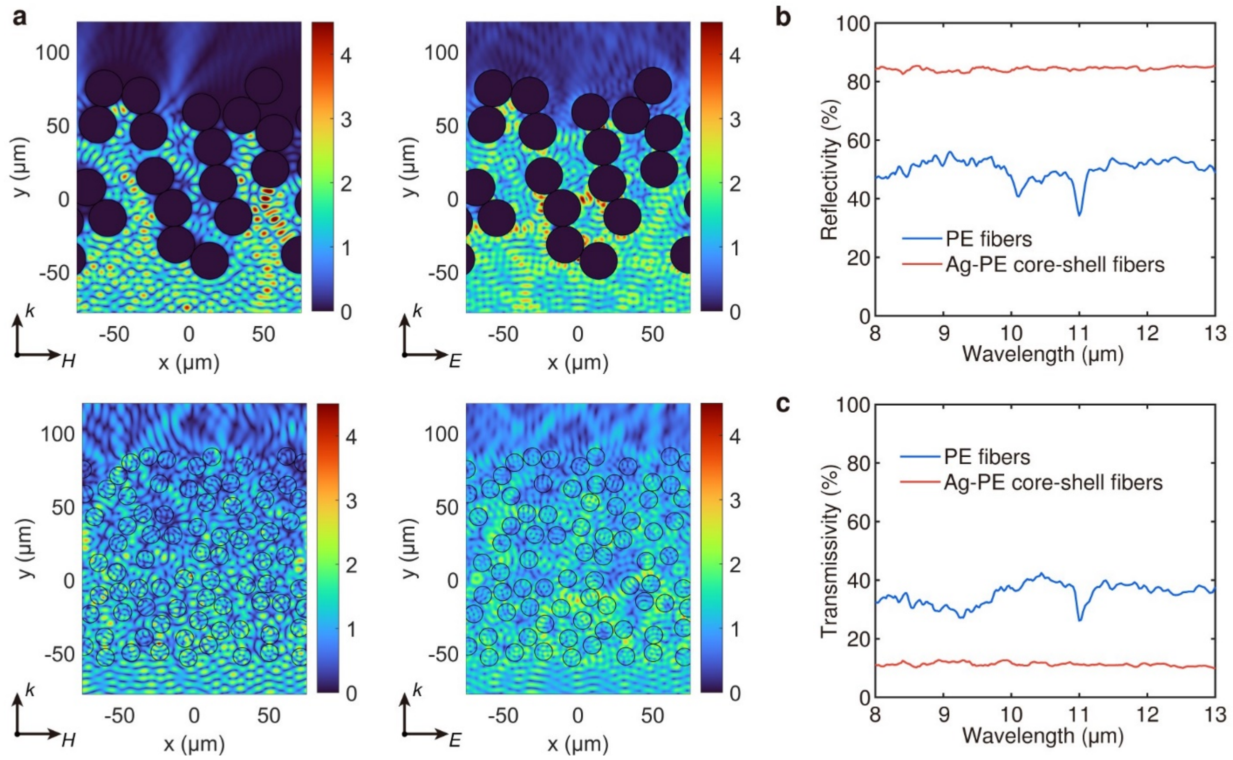

Figure S8. Calculated infrared reflective properties of Ag-PE core-shell fibers (LWIR shielding fiber) with diameter of 26.6 μm and pure PE fibers with diameter of 12 μm, related to Figure 2 and STAR methods. (a) Distribution of the simulated electric field of Ag-PE core-shell fibers and pure PE fibers for differently polarized incident light based on 2D FDTD simulations. Calculated infrared (b) reflectivity and (c) transmissivity of Ag-PE core-shell fibers and pure PE fibers. The Ag-PE core-shell fiber has stronger backscattering. The fibers are randomly distributed within a range of 150 μm (the thickness of LWIR shielding non-crimp fabric layer in the experiment). With limited thickness, Ag-PE core-shell fibers require less number of scattering events to reverse the incident light, resulting in higher reflectivity.

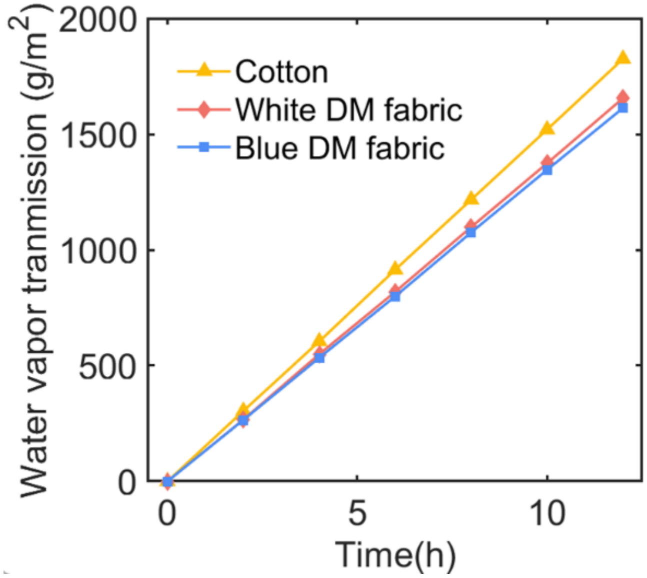

Figure S9. Water vapor transmission rates of different fabrics, related to Figure 2 and STAR methods. The results show that the DM fabrics have comparable water vapor transmission rates to commercial fabrics like cotton, implying their good breathability.

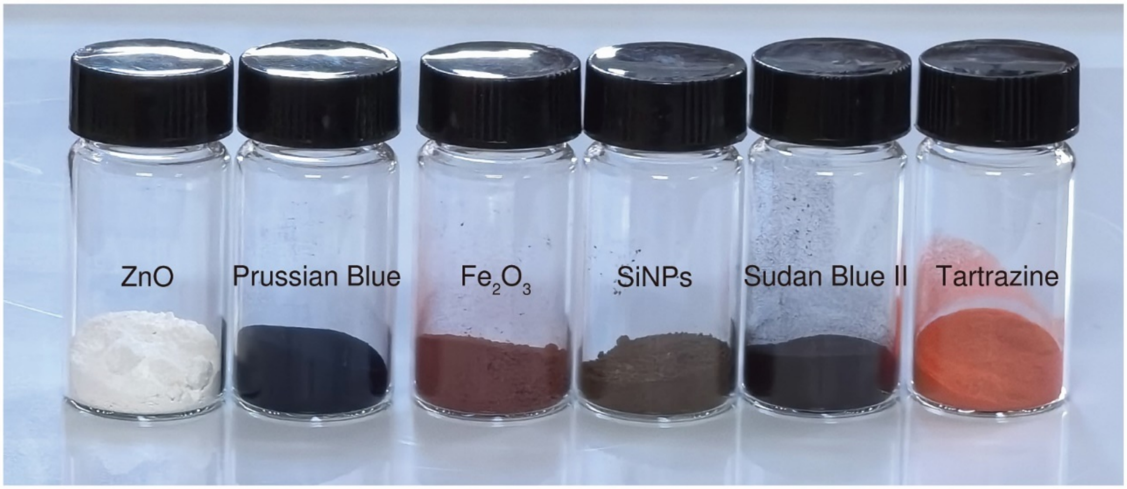

Figure S10. Photographs of dyes and pigments used to color the DM fabric, related to Figure 2 and STAR methods.

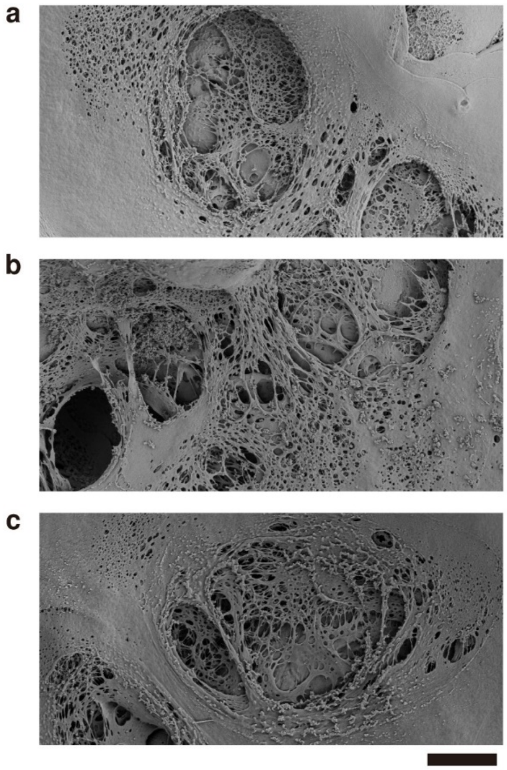

Figure S11. SEM images of (a) blue, (b) red and (c) yellow colored layers on the cooling side of the DM fabric, related to Figure 2. Scale bar, 8 μm. The porous structures of the colored layers help maintain the breathability of the DM fabric.

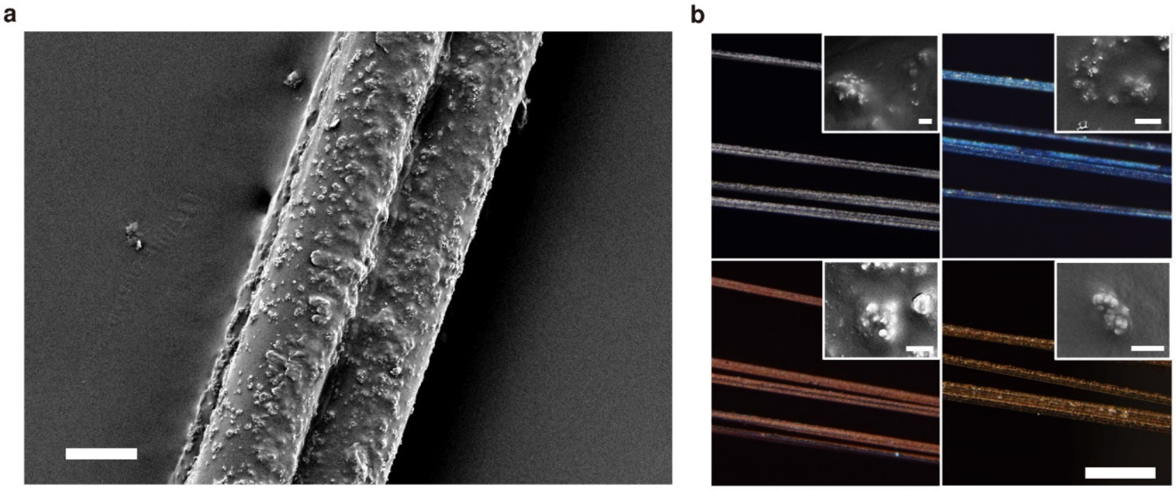

Figure S12. Morphology of LWIR shielding fibers, related to Figure 2. (a) SEM image of blue LWIR shielding fibers. PB was doped into the PE shell to achieve fiber coloring. Scale bar, 20 μm. (b) Optical micrographs of different colored LWIR shielding fibers Scale bar, 500 μm. Insets show SEM images of IRTPs doped in the PE shell of the fibers. Scale bars, 1 μm.

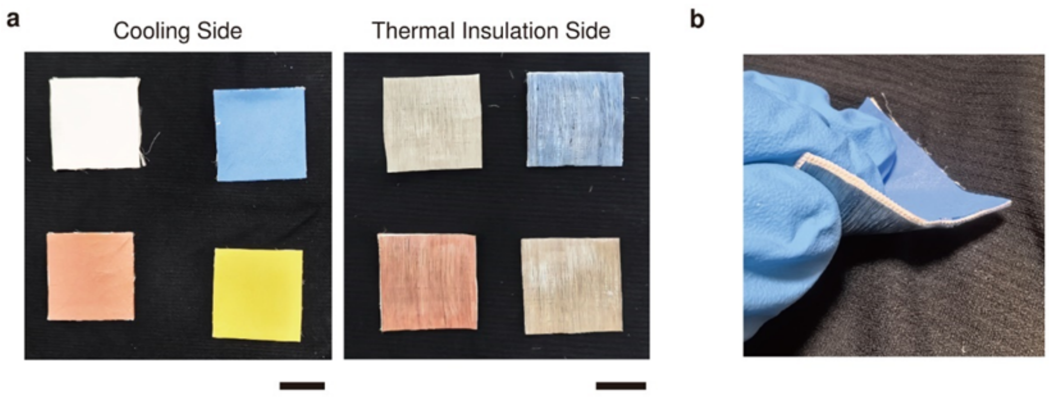

Figure S13. Photos of the DM fabrics with different colors, related to Figure 2. (a) Photos of the cooling and thermal insulation sides of the fabrics with different colors. Scale bars, 25 mm (b) Photo of the blue DM fabric as representative.

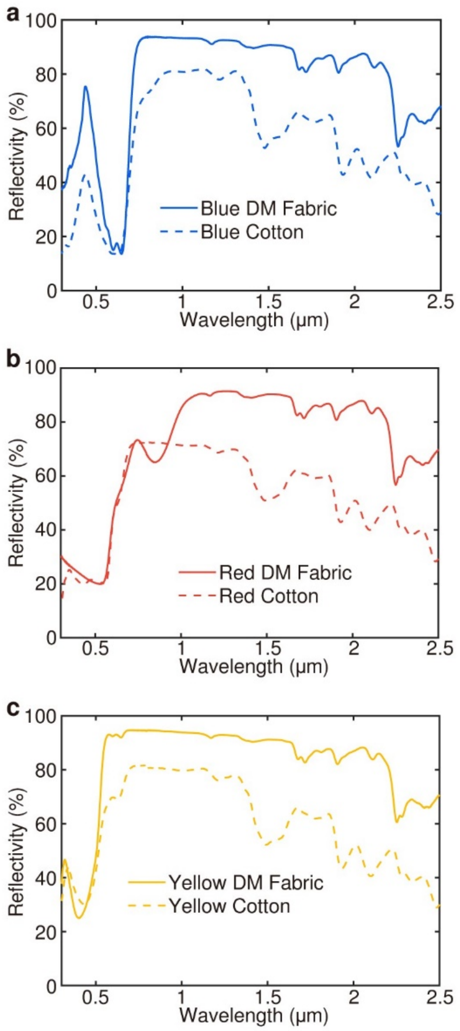

Figure S14. Solar radiation reflectivity of the cooling side of the DM fabrics and commercial cotton fabrics with (a) blue, (b) red and (c) yellow colors, related to Figure 2. The DM fabrics provide higher NIR reflection, minimizing the absorption of solar radiation while showing colors.

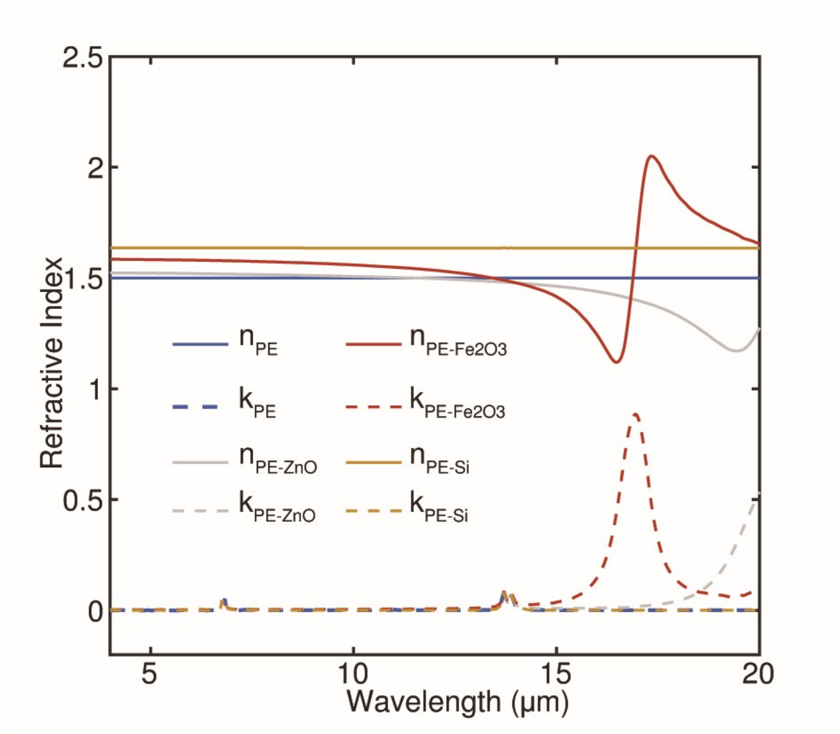

Figure S15. Calculated effective infrared optical indices of PE dopes with IRTPs with 10% volume ratio, related to Figure 2. The introduction of IRTPs hardly increases the absorption in the ATSW band.

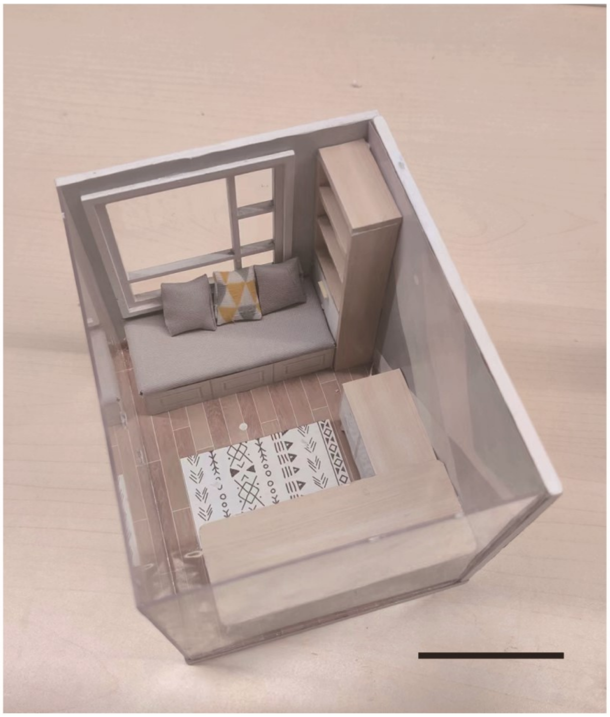

Figure S16. Photograph of a miniature house model used in the experiments, related to Figure 4. Scale bar, 5cm.

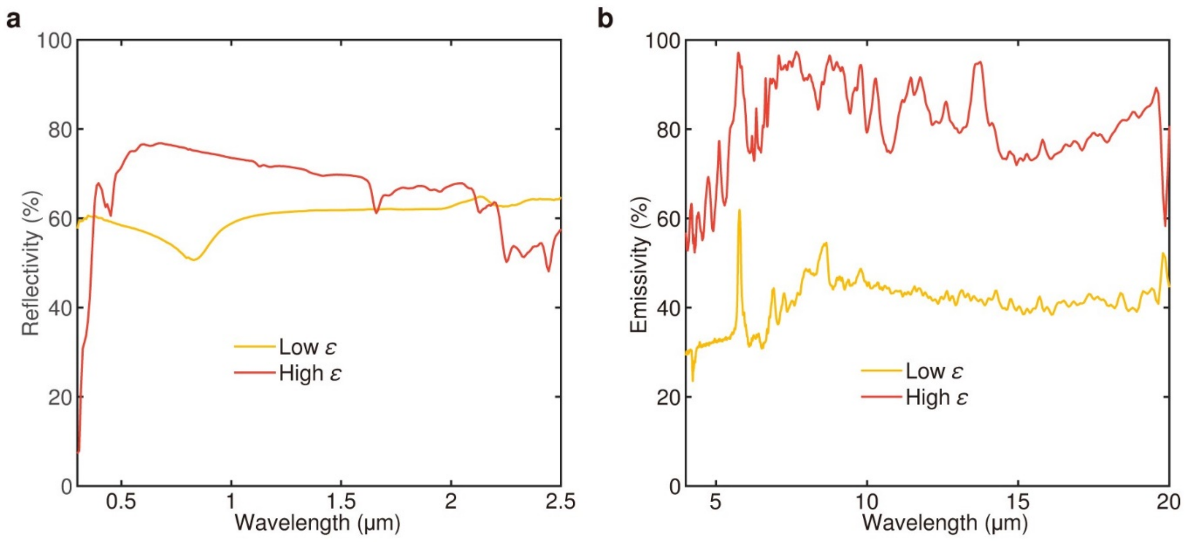

Figure S17. Optical properties of commercial shade cloths used in the experiments, related to Figure 4. (a) Reflectivity in the solar radiation band (0.3-2.5 μm). (b) Emissivity in the infrared band (4-20 μm).

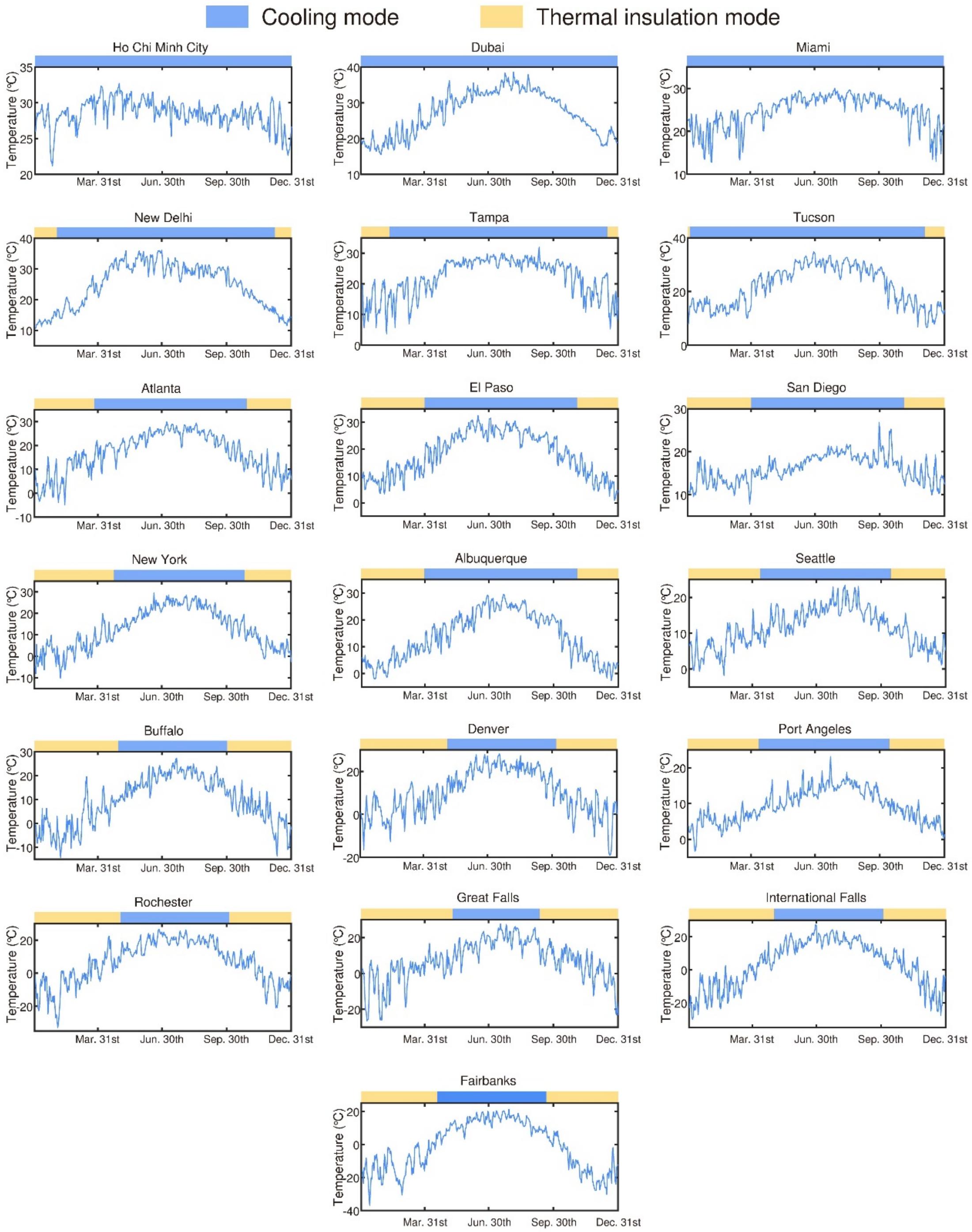

Figure S18. Average daily temperatures throughout the year for representative cities in different climate zones, as well as the corresponding operating mode of the DM fabric determined at a monthly frequency, related to Figure 5.

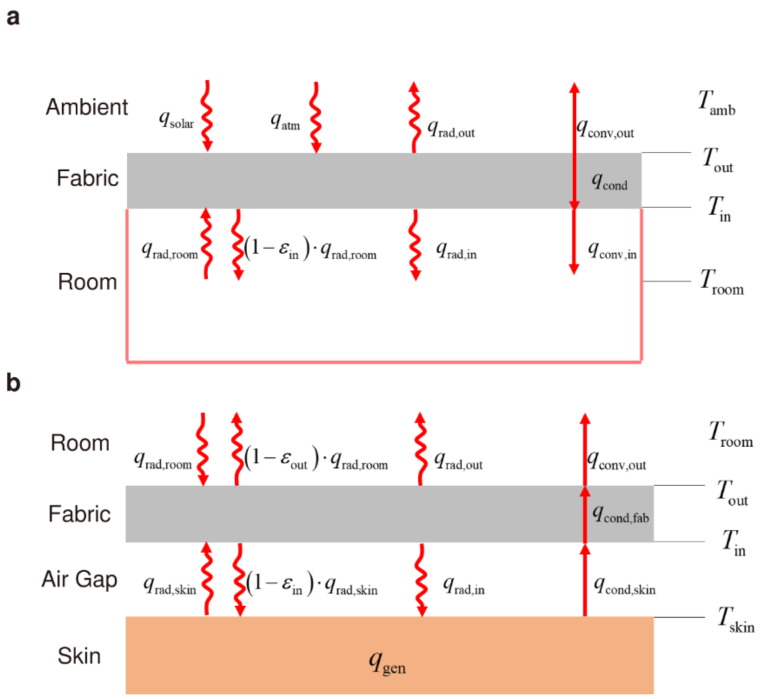

Figure S19. Schematics of the models used to calculated the net radiative heat flux of the DM fabric to a closed chamber (a) and the heating set points of HVAC system when a person wearing different fabrics (b), related to STAR methods.

Supporting Tables

Table S1. The thickness of each fabric layer, related to Figure 2

Cooling Side Colored Layer	UV Reflective Layer	Radiative Cooling Layer	LWIR Shielding non-crimp fabric Layer
~40 μm	~80 μm	~400 μm	~150 μm

Table S2. Parameters used in the heat transfer model for net heat flux to the indoor environment, related to STAR methods

Symbol	Definition	Value	Unit
I	Spectral radiance	$I_{AM1.5}$, AM1.5 solar spectrum<br>I_{BB}, blackbody thermal radiation	$W \cdot m^{-2} \cdot nm^{-1}$<br>$W \cdot sr^{-1} \cdot m^{-2} \cdot nm^{-1}$
T	Temperature	Room, T_{room} = 26<br>Ambient, T_{amb}<br>(for cooling, T_{amb}=35;<br>for Heating, T_{amb}=15)<br>Fabric inner surface, T_{in}<br>Fabric outer surface, T_{out}	°C
k	Thermal conductivity	Fabric, k_{fab} = 0.06	$W \cdot m^{-1} \cdot K^{-1}$
t	Thickness	Fabric, t_{fab} = 670	μm
σ	Stefan-Boltzmann constant	5.67×10^{-8}	$W \cdot m^{-2} \cdot K^{-4}$
ε	LWIR emissivity	Fabric inner surface, ε_{in}<br>(for low emissivity, ε_{in} =0.3;<br>for high emissivity, ε_{in} = 0.94)<br>Fabric outter surface, ε_{out}<br>(for low emissivity, ε_{out} =0.3;<br>for high emissivity, ε_{out} = 0.94)	N/A
α	Solar absorption	Fabric, α_{out}=0-1	N/A
h	Heat convection coefficient	Outer, h_{out} =8.3<br>Inner, h_{in} = 1	$W \cdot m^{-2} \cdot K^{-1}$

Table S3. Parameters used in the heat transfer model for indoor PTM, related to STAR methods

Symbol	Definition	Value	Unit
T	Temperature	Room, T_{room}<br>Fabric inner surface, T_{in}<br>Fabric outer surface, T_{out}<br>Skin, T_{skin}=34	°C
q_{gen}	Metabolic heat generation flux	101	W/m^2
k	Thermal conductivity	Fabric, k_{fab} = 0.064<br>Fabric, k_{air} = 0.026	$W \cdot m^{-1} \cdot K^{-1}$
t	Thickness	Fabric, t_{fab} = 670<br>Air, t_{fab} = 500	μm
σ	Stefan-Boltzmann constant	5.67×10^{-8}	$W \cdot m^{-2} \cdot K^{-4}$
ε	LWIR emissivity	Fabric inner surface, ε_{in}<br>Fabric outter surface, ε_{out}	N/A
h	Heat convection coefficient	Outer, h_{out} =4.1	$W \cdot m^{-2} \cdot K^{-1}$
